# Radiation Dose Optimization in Radiology: A Comprehensive Review of Safeguarding Patients and Preserving Image Fidelity

**DOI:** 10.7759/cureus.60846

**Published:** 2024-05-22

**Authors:** Sakshi S Dudhe, Gaurav Mishra, Pratapsingh Parihar, Devyansh Nimodia, Anjali Kumari

**Affiliations:** 1 Radiodiagnosis, Jawaharlal Nehru Medical College, Datta Meghe Institute of Higher Education and Research, Wardha, IND

**Keywords:** radiation radiology, radiation dose reduction, diagnostic imaging, alara principle, image fidelity, patient safety, radiology, radiation dose optimization

## Abstract

Radiation dose optimization in radiology is a critical aspect of modern healthcare, aimed at balancing the necessity of diagnostic imaging with the imperative of patient safety. This comprehensive review explores the fundamental principles, techniques, and considerations in optimizing radiation dose to safeguard patients while preserving image fidelity. Beginning with acknowledging the inherent risks associated with medical radiation exposure, the review highlights strategies such as the As Low as Reasonably Achievable (ALARA) principle, technological advancements, and quality assurance measures to minimize radiation dose without compromising diagnostic accuracy. Regulatory guidelines and the importance of patient education and informed consent are also discussed. Through a synthesis of current knowledge and emerging trends, the review underscores the pivotal role of radiation dose optimization in radiology practice. Furthermore, it emphasizes the need for ongoing research and collaboration to advance dose reduction strategies, establish standards for radiation safety, and explore personalized dose optimization approaches. By prioritizing radiation dose optimization, healthcare providers can ensure the highest standards of patient care while minimizing potential risks associated with medical radiation exposure.

## Introduction and background

In medical imaging, the optimization of radiation dose holds paramount significance. While diagnostic imaging techniques have revolutionized modern healthcare by enabling accurate diagnosis and treatment planning, they also entail exposure to ionizing radiation, which carries inherent risks [1]. Thus, optimizing radiation dose in radiology is essential to mitigate potential harm to patients while ensuring the acquisition of diagnostically valuable images [2].

Achieving the delicate equilibrium between patient safety and image quality is a fundamental objective in radiology practice. On the one hand, radiation is indispensable for obtaining clear and precise diagnostic images essential for effective clinical decision-making [3]. On the other hand, excessive radiation exposure can pose health risks, including potential carcinogenic effects. Therefore, radiologists and medical physicists are tasked with optimizing radiation doses to uphold patient safety without compromising diagnostic accuracy [4].

The primary aim of this comprehensive review is to delve into the multifaceted realm of radiation dose optimization in radiology. By synthesizing current knowledge, best practices, and emerging trends, this review intends to thoroughly understand the strategies and techniques employed to safeguard patients while preserving image fidelity. Through an exploration of fundamental principles, technological advancements, regulatory guidelines, and case studies, this review offers insights into the intricacies of radiation dose optimization and its pivotal role in modern radiology practice.

## Review

Fundamentals of radiation dose in radiology

Basic Principles of Radiation Dose

Radiation in medical imaging procedures must offer a more significant benefit to the patient than the potential risks associated with such exposure [5,6]. Imaging procedures must be conducted only when the anticipated medical advantages outweigh the potential risks from radiation. Consequently, efforts should be made to maintain radiation doses at levels that are "as low as reasonably achievable" (ALARA) without compromising the diagnostic quality of the resulting images [5,6]. Achieving this delicate balance requires concerted efforts from manufacturers, radiologists, technologists, and medical physicists to optimize equipment, protocols, and practices. While specific dose limits are not directly applicable to patients, they serve as guidelines for occupationally exposed workers to prevent unnecessary exposure to high radiation levels [6]. The International Commission on Radiological Protection (ICRP) recommends a practical dose limit of 20 mSv per year, averaged over five years, for radiation workers [6]. The foundational principles of radiation safety, namely time, distance, and shielding, play pivotal roles in minimizing radiation exposure [7]. Strategies such as reducing the duration of radiation exposure, maximizing distance from the radiation source, and employing appropriate shielding are essential in optimizing radiation dose [7]. In essence, these fundamental principles underpin collaborative efforts to balance image quality and radiation dose, safeguarding patients and preserving the fidelity of medical imaging [5,6].

Types of Radiation Used in Medical Imaging

**Figure 1:**
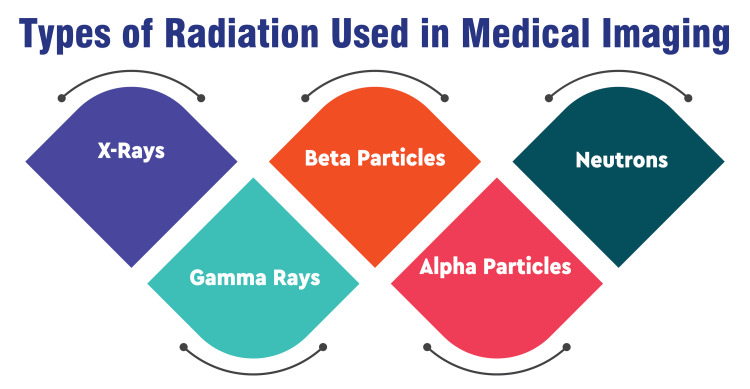
Types of radiation used in medical imaging Figure Credit: Dr Sakshi Dudhe

X-rays are a form of electromagnetic radiation characterized by high frequency and short wavelength, enabling them to penetrate the body and generate images of internal structures. Widely utilized in medical imaging, including techniques such as radiography, fluoroscopy, and computed tomography (CT) scans, X-rays play a fundamental role in diagnosing various medical conditions [8,9]. Gamma rays, another high-energy electromagnetic radiation, are emitted during radioactive decay. Possessing exceptionally short wavelengths and high penetrating power, gamma rays find application in nuclear medicine imaging modalities such as positron emission tomography (PET) and single-photon emission computed tomography (SPECT) [9]. Beta particles, comprising high-energy electrons emitted during radioactive decay, exhibit greater penetrating power than alpha particles. Although less commonly employed, beta particles are utilized in certain nuclear medicine procedures for diagnostic purposes [9]. On the other hand, alpha particles consist of two protons and two neutrons and are emitted by heavy radioactive elements during decay. Despite their low penetrating power, alpha particles pose significant hazards if inhaled or ingested. While not typically used in medical imaging, their potential health risks underscore the importance of proper handling and containment measures [9]. Types of radiation used in medical imaging are shown in Figure 1.

Factors Influencing Radiation Dose in Radiology

Numerous factors contribute to the radiation dose administered during radiology procedures, encompassing both patient-specific characteristics and procedure-related variables. Patient attributes such as gender, age, body composition, and prior radiation exposure significantly influence radiation dose levels encountered during imaging procedures [10-12]. Research indicates that men receive higher radiation doses in specific medical imaging contexts than women [10,12]. Furthermore, age disparities correlate with variations in radiation dose, with younger individuals typically receiving lower doses than their older counterparts [12]. Body composition, particularly in individuals with morbid obesity, can necessitate higher radiation doses to achieve satisfactory imaging outcomes [12]. Conversely, factors specific to the imaging procedure, such as its complexity, equipment settings, and the technique employed by the radiologist or radiographer, also impact radiation dose in radiology [13]. For instance, parameters including the number of lesions treated, stents placed, tortuosity grade, and occlusion stage in percutaneous coronary intervention (PCI) procedures have been identified as contributing factors to heightened patient radiation dose [10]. Moreover, the design characteristics of the CT scanner, slice collimation, and the utilization of advanced technologies such as iterative reconstruction algorithms can significantly influence radiation dose levels in CT examinations [13].

Risks and benefits of medical radiation exposure

Potential Risks Associated With Medical Radiation Exposure

The potential risks associated with medical radiation exposure encompass several adverse effects, including a slight elevation in the risk of developing cancer later in life, tissue-related effects such as cataracts, skin reddening, and hair loss, as well as potential reactions to contrast agents utilized in imaging procedures [8,14]. Ionizing radiation, commonly employed in medical imaging, possesses sufficient energy to induce DNA damage, thereby initiating cell mutations, chromosome translocations, and gene fusions implicated in cancer onset [14]. While the likelihood of developing cancer from radiation exposure in medical imaging is generally minimal, this risk escalates with higher radiation doses, increased frequency of X-ray examinations, younger age at exposure, and exposure to specific body regions [8]. Adherence to the ALARA principle is paramount for healthcare providers to mitigate radiation exposure while ensuring that the benefits derived from the imaging procedure outweigh the associated risks [8]. Particular attention should be directed towards pediatric patients and pregnant women, given their heightened sensitivity to radiation exposure [8]. Achieving a delicate balance between the advantages of precise diagnosis and treatment guidance and the potential hazards of radiation exposure is imperative in medical imaging practices, underscoring patient safety and well-being prioritization.

The Benefits of Diagnostic Imaging Outweigh the Risks

Medical imaging tests such as X-rays, CT scans, and fluoroscopy are pivotal in furnishing doctors with crucial information for diagnosing diseases, guiding treatments, and monitoring patient health [15,16]. Consequently, these examinations offer significant benefits, including enhanced patient outcomes and reductions in morbidity and mortality rates [16]. Despite small risks associated with medical radiation exposure, such as a slight elevation in lifetime cancer risk, these risks are typically outweighed by the substantial advantages conferred by the imaging procedure [15,17]. The risk of forgoing a necessary imaging test often exceeds the minimal radiation risk [15]. Healthcare providers adhere to the ALARA principle to minimize radiation exposure while ensuring the acquisition of essential diagnostic information [15,16]. This principle encompasses utilizing the lowest radiation dose settings, implementing shielding measures, and considering alternative imaging modalities such as ultrasound or MRI when appropriate [16]. Furthermore, special considerations are extended to higher-risk populations, such as children and pregnant women, focusing on further reducing radiation exposure while maintaining essential medical care [15,16].

Understanding Radiation Dose Limits and Thresholds

The ICRP recommends dose limits to safeguard individuals from unnecessary exposure to ionizing radiation [18]. For the general public, the effective dose limit is set at one mSv per year, with allowances for higher values if the average over five years does not exceed one mSv per year [18,19]. Occupationally exposed workers have a higher limit of 20 mSv per year, averaged over five years [18]. Notably, these dose limits do not pertain to medical exposures, as radiation exposure in medical settings is intentional and aimed at directly benefiting the patient. Instead, the focus lies on justifying medical procedures and optimizing radiation protection by applying the ALARA principle [18,20]. Diagnostic reference levels (DRLs) are valuable tools for optimizing radiation protection within medical imaging. These levels represent the upper thresholds (75th percentile) of typical radiation doses for standard medical imaging examinations [21]. Deterministic radiation effects, such as tissue damage, exhibit clear dose thresholds. For instance, the threshold for acute radiation effects like nausea and vomiting is approximately 1,000 mSv, while skin reddening typically occurs at doses around 2,000 mSv [19]. The linear no-threshold (LNT) model forms the basis for radiation safety practices, positing that any radiation dose, irrespective of magnitude, carries a risk of stochastic effects such as cancer. However, ongoing debate persists regarding the validity of the LNT model, particularly concerning its applicability to low radiation doses encountered in medical imaging [22].

Techniques for radiation dose optimization

ALARA Principle

The ALARA principle stands as a cornerstone in radiation protection, advocating for minimizing radiation exposure to humans, animals, or materials while ensuring that image quality remains adequate for diagnosis [23]. This principle is a linchpin in radiation protection programs and regulatory frameworks, guiding all endeavours to diminish radiation exposure while acknowledging the inherent risks associated with ionizing radiation [24]. At the core of ALARA lies the conservative assumption that any level of radiation dose, irrespective of its magnitude, has the potential to induce detrimental effects, such as an elevated risk of genetic mutations and cancer, as dictated by the linear hypothesis model of radiation dose and its biological impact on living tissues [25]. Effectively implementing ALARA necessitates focusing on three primary safety principles: time, distance, and shielding [26]. Reducing the duration of exposure directly mitigates radiation dose while increasing the distance between individuals and radiation sources exponentially decreases exposure levels. Moreover, employing appropriate shielding materials, such as lead for X-rays and gamma rays, further diminishes radiation exposure [26]. In addition to these principles, adherence to good hygiene practices, contamination control, avoidance of airborne hazards, and using proper personal protective equipment (PPE) are indispensable in curtailing internal radiation exposure [26]. By diligently applying these measures, radiation exposure can be minimized while ensuring the safety and well-being of individuals and the environment.

Equipment and Technology Advancements

Manufacturers have developed dose management technologies to enhance radiation safety during interventional procedures. These technologies utilize X-ray skin colour-coded indicators on a 3D visual representation of the patient, enabling clinicians to make real-time adjustments to minimize the risk of locally concentrated high radiation exposure [27]. Flat-panel digital detectors represent another significant advancement in dose efficiency within modern imaging systems. Coupled with advanced image processing techniques, these detectors reduce radiation doses for patients and operators. Notably, in chest imaging, flat-panel detectors have been demonstrated to improve image quality while lowering radiation exposure [27]. In CT imaging, iterative reconstruction technologies have revolutionized dose reduction while preserving essential image quality and anatomical detail. By delivering noise reduction, resolution enhancement, improved contrast, and artefact suppression, these technologies facilitate the administration of reduced radiation doses, which is particularly beneficial for vulnerable patient groups such as pediatric patients and women of childbearing age [28]. Advanced automatic exposure control (AEC) technologies, including real-time 3D dose modulation, play a vital role in ensuring consistent image quality by dynamically adjusting exposure parameters during imaging procedures. Moreover, dynamic Z-axis tracking reduces unnecessary radiation in helical scanning by automatically correcting the X-ray beam position to block unused radiation at the beginning and end of a scan [29]. In interventional cardiology, prospective triggered gating technology is a promising innovation for minimizing patient radiation exposure. By activating the X-ray only during critical phases of the cardiac cycle, this technology significantly reduces X-ray exposure time, with studies indicating potential reductions of up to 70% [30].

Image Acquisition Parameters Optimization

The optimization of image acquisition parameters in medical imaging plays a pivotal role in striking a delicate balance between radiation dose, image quality, and diagnostic accuracy. Numerous studies and research articles underscore the importance of optimizing these parameters to yield high-quality images while minimizing radiation exposure. Critical aspects of image acquisition parameter optimization encompass adjusting factors such as kVp, mAs, signal-to-noise ratio (SNR) levels, and iterative reconstruction techniques to enhance image quality and reduce noise levels [31]. Moreover, carefully selecting acquisition times, reconstruction algorithms, and post-processing filters significantly optimize image acquisition parameters across various imaging modalities, including CT and SPECT/CT [31,32]. Research indicates that optimizing acquisition parameters entails striking the right balance between radiation dose and image quality. For instance, in CT brain protocols, recommended parameters include setting kVp between 100 and 120, maintaining mAs within a range of 200 to 300, ensuring SNR levels fall within specific thresholds, and employing iterative reconstruction techniques to achieve optimal image quality while effectively managing radiation dose [31]. Furthermore, adopting advanced technologies such as adaptive statistical and hybrid iterative reconstruction algorithms has substantially impacted image quality in CT imaging, facilitating dose reduction without compromising diagnostic efficacy [31].

Patient-Specific Considerations

The hospital management holds a paramount responsibility to prevent unintended and accidental medical exposures, and in cases where such exposures occur, they must be promptly investigated and corrective actions implemented [33]. These exposures encompass medical treatment administered to the wrong individual or organ, exposures substantially exceeding intended levels, and inadvertent exposure of the embryo or fetus [33]. Particular attention should be given to vulnerable patient populations to optimize their protection and safety during medical procedures [33]. This includes pediatric patients, pregnant individuals, and those undergoing repeated imaging procedures, for whom tailored protective measures are essential. Justifying radiological procedures is integral to ensuring their necessity and appropriateness for individual patients [34]. Referring medical practitioners are responsible for carefully evaluating whether a radiological procedure is warranted, whether it represents the best investigation to address the clinical question, and whether the clinical problem has been adequately communicated to the radiological medical practitioner. This meticulous evaluation helps justify and optimize radiological procedures for each patient's circumstances. In optimizing protection and safety, dose constraints should be applied to individuals serving as carers or comforters and those exposed to biomedical research programs [35]. These constraints aim to minimize radiation exposure for these individuals while ensuring their well-being. Established DRLs are recommended for the most common diagnostic procedures to aid in optimizing patient radiation dose [35]. By adhering to these reference levels, healthcare providers can standardize radiation doses while maintaining diagnostic efficacy and patient safety.

Quality Assurance (QA) and Control Measures

Implementation of QA programs: Establishing QA programs within radiology is paramount to upholding diagnostic imaging procedures' reliability, safety, and efficacy. These QA programs encompass various facets of the imaging process, spanning equipment checks, pre-procedural preparations, and routine quality control assessments conducted regularly. Their overarching objective is to oversee the entire imaging system, ensuring proper image acquisition and processing while maintaining established image quality standards [36]. Implementing QA programs entails meticulous planning, ongoing operation, and allocation of resources in terms of time and finances [36]. Its primary aim is to furnish adequate clinical diagnostic information while minimizing patient exposure to radiation, adhering to the principle of ALARA [36]. QA programs extend beyond mere compliance with legal requirements concerning the quality control of X-ray equipment. They also underscore the optimal utilization of equipment, human, and material resources. This is achieved through initiatives such as film rejection analysis and the monitoring of patient radiation doses during radiological examinations [36]. In diagnostic radiology, implementing QA programs is indispensable for optimizing radiological practices, aligning with international standards, and safeguarding patient well-being. These programs necessitate the development of documented QA protocols tailored to the unique needs of individual departments, delineating items for monitoring and establishing testing intervals [37]. The involvement of qualified medical physicists is pivotal at every stage of QA program development, from conception to evaluation, for their success and efficacy [37]. Their expertise is instrumental in designing, initiating, implementing, and assessing QA initiatives, ensuring these programs are robust, comprehensive, and aligned with enhancing radiological practices and patient safety.

Regular calibration and maintenance of imaging equipment: They are indispensable in ensuring diagnostic results' accuracy and reliability within medical imaging practices. Calibration entails aligning or adjusting a device to match a known standard, ensuring its readings are precise and consistent [38]. This process is crucial for detecting and rectifying any measurement deviations that could compromise diagnostic accuracy and patient outcomes [39]. In contrast, maintenance focuses on identifying and rectifying faults within devices to ensure their proper functioning. By conducting regular calibration and maintenance procedures, errors can be preemptively prevented, accuracy can be preserved, and the lifespan of imaging equipment can be extended, thereby safeguarding patient safety and optimizing diagnostic procedures [40]. Various imaging equipment, such as CT scanners, necessitate routine calibration to uphold image accuracy, prevent distortion, and maintain appropriate contrast levels for accurate diagnoses [41]. During calibration, parameters such as CT number, linearity, uniformity, spatial resolution, noise, low contrast resolution, and slice thickness are meticulously tested to ensure optimal imaging quality [41]. Through diligent calibration and maintenance efforts, healthcare providers can uphold the integrity and efficacy of medical imaging practices, thereby enhancing patient care and treatment outcomes.

Regulatory Guidelines and Standards

Overview of international radiation safety guidelines: The International Atomic Energy Agency (IAEA) has formulated a comprehensive framework of international safety standards aimed at safeguarding radiation protection and ensuring the safety of radiation sources, known as the International Basic Safety Standards (BSS) [42-44]. Grounded in the fundamental safety principles outlined in the IAEA Safety Fundamentals (SF-1), these standards protect individuals and the environment from the adverse effects of ionizing radiation while avoiding undue limitations on operating facilities or activities [42,43]. Sponsored jointly by several international organizations, including the World Health Organization (WHO), Food and Agriculture Organization (FAO), International Labour Organization (ILO), and Organisation for Economic Co-operation and Development/Nuclear Energy Agency (OECD/NEA), the BSS standards are informed by the scientific findings and recommendations of bodies such as United Nations Scientific Committee on the Effects of Atomic Radiation (UNSCEAR) and ICRP [42-44]. The foundational principles of the ICRP system of radiation protection - justification, optimization, and dose limitation - serve as cornerstones for the BSS standards [45]. These standards delineate requirements for protecting individuals and the environment, with accompanying safety guides offering recommendations on how to adhere to these requirements [45]. While the IAEA safety standards are not legally binding, they represent a global consensus on optimal practices for ensuring a high level of safety. They are widely adopted by national regulatory bodies and organizations involved in radiation-related endeavours [45].

Compliance With Regulatory Requirements

Ensuring compliance with regulatory requirements in the field of radiological protection is paramount to upholding the safety of individuals exposed to ionizing radiation. The IAEA highlights the significance of adhering to radiation protection principles outlined in the BSS, which encompass the justification of practices, dose limitation, and optimization of protection and safety [46]. These principles are guiding pillars for establishing dose constraints, particularly in diagnostic medical exposure, where optimizing protection entails minimizing patient exposure while achieving diagnostic objectives [46]. In the United Kingdom, the Ionising Radiations Regulations 2017 delineate specific dose limits for members of the public and workers, laying out legal mandates to ensure radiation exposure remains within permissible levels [47]. Furthermore, these regulations stipulate dose constraints for radioactive discharge regulation, underscoring the imperative of maintaining doses below specified thresholds to safeguard individuals and the environment [47]. Compliance with these dose limits and constraints is indispensable to prevent radiation exposure from surpassing acceptable levels and to uphold the public's and workers' health and safety in facilities utilizing radioactive substances. Employers in medical settings are subject to The Ionising Radiation (Medical Exposure) Regulations 2017, which mandate the collection of dose estimates from medical exposures, establishment of dose constraints for research programs, and prompt investigation of any accidental or unintended exposures [48]. These regulations underscore the criticality of maintaining awareness regarding the effects of ionizing radiation, conducting clinical audits, informing individuals exposed to radiation, and implementing QA programs to ensure compliance with regulatory requirements [48]. Compliance with regulatory requirements in radiological protection necessitates a comprehensive approach that encompasses justifying practices, setting dose limits and constraints, optimizing protection, and ensuring the implementation of proper procedures to monitor, investigate, and mitigate potential risks associated with ionizing radiation exposure.

Advanced imaging modalities and dose reduction strategies

Introduction to Advanced Imaging Techniques

Iterative reconstruction represents a cutting-edge CT technology that empowers clinicians to diminish radiation dose without compromising image quality or anatomical detail [49]. Products utilizing iterative reconstruction offer superior noise reduction, resolution enhancement, contrast improvement, and artefact suppression compared to traditional filtered back-projection methods. AEC systems, such as real-time 3D dose modulation, contribute to consistent image quality by automatically adjusting the X-ray tube current based on patient size and anatomy [27,49]. This ensures that the optimal radiation dose is administered to each patient, enhancing safety and efficacy. Dynamic Z-axis tracking, employed in helical CT scanning, facilitates automatic and continuous correction of the X-ray beam position throughout the scan. This functionality effectively blocks unused radiation at the start and end of the scan, thereby reducing unnecessary dose exposure [27]. In cardiac CT imaging, prospective triggered gating technology selectively activates the X-ray solely during critical phases of the cardiac cycle, substantially reducing up to 70% in a patient's radiation exposure time [27]. Spectral filtration is a feature that allows for programmable filtration of the X-ray beam, ensuring accurate delivery of the radiation beam tailored to a specific procedure's specific requirements. This optimization of dose utilization enhances the efficacy and safety of the imaging process [27]. Specialized pediatric imaging protocols, pre-loaded on equipment, are pivotal in reducing radiation exposure in children while preserving high-quality diagnostic images [27]. These advanced techniques, comprehensive staff training, and adherence to the ALARA principle empower radiology departments to optimize radiation dose across a broad spectrum of imaging modalities and clinical applications [50,51].

Emerging Technologies and Future Directions

Emerging technologies in radiology are reshaping the landscape of medical imaging, offering promising advancements that hold the potential to revolutionize patient care. Artificial intelligence (AI) stands at the forefront, fundamentally transforming radiology by augmenting image analysis, facilitating diagnoses, and enhancing patient outcomes through automation and predictive analytics [52,53]. Dark field radiography, an innovative technology that focuses on the properties of X-ray waves, presents the opportunity for improved visualization of soft tissues and structures, potentially revolutionizing chest radiography and MRI/CT imaging [54]. Radiomics, a burgeoning field within radiology, extracts quantitative features from medical images to personalize patient care, predict treatment outcomes, and deepen our understanding of diseases [55]. Robotic systems are increasingly vital in radiology, offering precise imaging and reduced radiation exposure, thereby revolutionizing procedures and optimizing patient positioning [55]. Augmented reality (AR) technology is augmenting radiologists' capacity to interact with medical images, enhancing accuracy in interventional procedures and enriching medical education [55]. Moreover, advancements in MRI technology render it more accessible, cost-effective, and user-friendly, with enhanced image quality and accelerated scan times. This progress drives its adoption as a primary imaging modality in clinical settings [55]. These technologies signify the future trajectory of radiology, promising more precise diagnoses, improved patient care, and heightened efficiency in medical imaging practices.

Patient Education and Informed Consent

In medical imaging, patient education regarding radiation risks is paramount to ensure individuals are adequately informed about the benefits and potential hazards of radiation exposure. Numerous studies underscore the critical role of patient education in mitigating uncertainty, anxiety, and fear related to radiation exposure, thereby empowering patients to make well-informed decisions about their healthcare [56-58]. Effective patient education enhances patient satisfaction and compliance and fosters patient empowerment, encouraging active involvement in their care and ultimately improving treatment outcomes and adherence [59]. Communicating radiation risks to patients in clear and understandable terms is essential, as well as addressing their knowledge, beliefs, and attitudes toward radiation exposure sources to facilitate informed decision-making and alleviate concerns [58]. Furthermore, patient education assumes heightened importance in promoting active patient participation, particularly within the evolving healthcare landscape where patients are increasingly engaged in treatment decisions [60]. By furnishing comprehensive information about radiation risks, benefits, and potential side effects, patient education empowers individuals to comprehend the implications of medical imaging procedures and make informed choices about their healthcare journey [61].

Case studies and clinical examples

Real-World Examples of Radiation Dose Optimization Techniques

AEC encompasses techniques such as automatic current modulation and automatic current selection, which optimize radiation dose by adjusting exposure parameters based on patient anatomy and diagnostic requirements [62]. This adaptive approach ensures that the appropriate amount of radiation is administered, tailored to each patient's specific needs, thereby enhancing safety and efficacy. Iterative reconstruction technologies represent another advancement in dose reduction strategies, enabling the preservation of image quality and anatomical detail while reducing radiation exposure. Particularly advantageous for vulnerable patient groups such as children and women of childbearing age, these technologies significantly improve patient safety and diagnostic accuracy [27]. Advanced adaptive image filters play a pivotal role in dose reduction efforts by identifying image features and making processing adjustments to reduce dose while maintaining image quality. In cardiac imaging, where the preservation of coronary anatomy is paramount, these filters are instrumental in achieving optimal outcomes with minimal radiation exposure [27]. AEC in cardiac CT leverages technologies like real-time 3D dose modulation and dynamic Z-axis tracking to deliver consistent image quality while minimizing unnecessary radiation exposure, especially in cardiac CT imaging. These innovations ensure precise imaging outcomes while safeguarding patient health [27]. Interventional X-ray dose reduction techniques revolutionize interventional procedures by enabling greater precision while minimizing unnecessary radiation exposure. Innovations such as spot fluoroscopy enhance imaging with lower doses, enhancing patient safety and procedural efficacy [27]. Pediatric imaging techniques encompass various strategies to reduce radiation exposure in children while maintaining high-quality images significantly. Utilizing pre-loaded pediatric protocol selection tools, removable grids, and child-friendly equipment, these techniques optimize dose in pediatric imaging, prioritizing patient safety and diagnostic accuracy [62].

Impact of Dose Reduction Strategies on Image Quality and Diagnostic Accuracy

Dose reduction strategies in radiology are pivotal for striking a balance between radiation exposure, image quality, and diagnostic precision. By optimizing radiation doses, healthcare professionals can achieve the crucial dual objective of minimizing patient exposure to ionizing radiation while ensuring high-quality diagnostic images conducive to accurate interpretation. Various techniques, including tube current modulation, kVp modulation, scan length adjustment, dynamic z-axis collimation, iterative reconstruction, and dual-energy imaging, are employed to curtail radiation doses while upholding image quality [27,63]. These strategies empower clinicians to tailor CT acquisition parameters according to clinical information and patient demographics, significantly reducing radiation doses without compromising diagnostic accuracy [63]. Moreover, advancements in dose reduction technologies, such as adaptive image filters and AEC, contribute to maintaining image quality while mitigating radiation exposure in cardiac imaging and other procedures [63]. Implementing these dose optimization strategies bolsters patient safety by mitigating radiation risks and ensures that medical imaging remains a dependable tool for precise diagnosis and treatment planning.

Patient Outcomes and Experiences

Patient outcomes and experiences in radiation dose optimization in radiology are pivotal considerations influenced by the strategies and technologies implemented to safeguard patients while upholding image fidelity. Medical imaging professionals can strike a delicate balance between diagnostic accuracy and patient safety by prioritizing the optimization of radiation dose through innovations such as iterative reconstruction technologies, dose reduction programs, and adaptive image filters [27,50]. These advancements diminish radiation exposure for patients, especially vulnerable groups like children and individuals requiring frequent follow-up scans, and bolster image quality and anatomical detail, resulting in more precise diagnoses and treatment planning [27,50]. Furthermore, integrating dose reduction technologies in interventional X-ray procedures facilitates enhanced precision during medical interventions, minimizing unnecessary radiation exposure and ultimately improving patient outcomes [27]. Patient experiences are further enriched through innovative techniques such as spot fluoroscopy, which offers heightened imaging precision with reduced doses, enabling quicker diagnoses and mitigating patient discomfort during procedures [27]. Additionally, introducing child-friendly equipment and pre-loaded pediatric protocols contributes to cultivating a more comfortable environment for pediatric patients, eliminating the need for sedation and additional imaging, thus augmenting their overall experience and compliance with medical procedures [27].

## Conclusions

In conclusion, this comprehensive review underscores the importance of radiation dose optimization in radiology. Throughout our exploration, we have emphasized the need to balance patient safety with the imperative of maintaining high-quality diagnostic images. By adhering to principles such as ALARA and leveraging technological advancements, healthcare providers can minimize radiation exposure while maximizing diagnostic accuracy. Furthermore, implementing robust QA measures and adherence to regulatory guidelines are essential in ensuring consistent radiation safety standards across healthcare settings. Looking ahead, ongoing research efforts and technological innovations will continue to shape the landscape of radiation dose optimization. Future endeavours may focus on refining dose reduction strategies, exploring personalized approaches to dose optimization, and elucidating the long-term effects of cumulative radiation exposure. By remaining vigilant and proactive in our pursuit of radiation safety, we can uphold the highest standards of patient care while advancing the field of radiology.

## References

[1] Najjar R (2023). Radiology’s ionising radiation paradox: weighing the indispensable against the detrimental in medical imaging. Cureus.

[2] Akram S, Chowdhury YS (2023). Radiation exposure of medical imaging. StatPearls [Internet].

[3] European Society of Radiology (ESR); European Federation of Radiographer Societies (EFRS) (2019). Patient safety in medical imaging: a joint paper of the European Society of Radiology (ESR) and the European Federation of Radiographer Societies (EFRS). Insights Imaging.

[4] (2024). Radiation in healthcare: imaging procedures. https://www.cdc.gov/radiation-health/features/imaging-procedures.html.

[5] (2024). Radiation dose. https://www.radiologyinfo.org/en/info/safety-xray.

[6] Murphy A (2024). Radiation protection. https://radiopaedia.org/articles/radiation-protection?lang=gb.%2010.53347/rID-52566.

[7] (2024). Radiation safety principles and governance. https://www.boa.ac.uk/standards-guidance/radiation-exposure-in-theatre/t-and-o/radiation-safety-principles-and-governance.html.

[8] Health C for D and R (2024). Medical X-ray imaging. https://www.fda.gov/radiation-emitting-products/medical-imaging/medical-x-ray-imaging.

[9] Donya M, Radford M, ElGuindy A, Firmin D, Yacoub MH (2014). Radiation in medicine: origins, risks and aspirations. Glob Cardiol Sci Pract.

[10] Tsapaki V, Magginas A, Vano E (2006). Factors that influence radiation dose in percutaneous coronary intervention. J Interv Cardiol.

[11] Parry RA, Glaze SA, Archer BR (1999). The AAPM/RSNA physics tutorial for residents. Typical patient radiation doses in diagnostic radiology. Radiographics.

[12] Nagpal P, Priya S, Eskandari A (2020). Factors affecting radiation dose in computed tomography angiograms for pulmonary embolism: a retrospective cohort study. J Clin Imaging Sci.

[13] Nagel HD (2007). Ct parameters that influence the radiation dose. Radiation Dose from Adult and Pediatric Multidetector Computed Tomography. Springer: Berlin, Heidelberg.

[14] Lin EC (2010). Radiation risk from medical imaging. Mayo Clin Proc.

[15] (2024). Medical imaging: what you need to know. https://www.gov.uk/government/publications/medical-imaging-what-you-need-to-know/medical-imaging-what-you-need-to-know--2.

[16] (2024). Benefits and risks of medical imaging tests. https://www.lompocvmc.com/blogs/2019/november/benefits-and-risks-of-medical-imaging-tests/.

[17] Zanzonico PB (2019). Benefits and risks in medical imaging. Health Phys.

[18] Murphy A (2024). Dose limits. https://radiopaedia.org/articles/dose-limits?lang=gb.%2010.53347/rID-53065.

[19] (2024). Limit values in radiation protection. https://www.bfs.de/EN/topics/ion/radiation-protection/limit-values/limit-values_node.html.

[20] Matsubara K (2021). Assessment of radiation dose in medical imaging and interventional radiology procedures for patient and staff safety. Diagnostics (Basel).

[21] (2024). How to understand and communicate radiation risk. https://www.imagewisely.org/Imaging-Modalities/Computed-Tomography/How-to-Understand-and-Communicate-Radiation-Risk.

[22] Moorthy S (2021). How safe are radiation doses in diagnostic radiology? A historical perspective and review of current evidence. Indian J Radiol Imaging.

[23] Gerstmair A (2024). As low as reasonably achievable (ALARA). https://radiopaedia.org/articles/as-low-as-reasonably-achievable-alara?lang=gb.%2010.53347/rID-35183.

[24] Frane N, Bitterman A (2023). Radiation safety and protection. StatPearls [Internet].

[25] Committee on the Analysis of Cancer Risks in Populations near Nuclear Facilities-Phase I; Nuclear and Radiation Studies Board; Division on Earth and Life Studies; National Research Council (2012). Analysis of Cancer Risks in Populations Near Nuclear Facilities: Phase I. https://www.ncbi.nlm.nih.gov/books/NBK201996/.

[26] Jaquith K: 7 ALARA Principles For Reducing Radiation Exposure (2024). 7 ALARA principles for reducing radiation exposure. Universal Medical Inc. Blog.

[27] (2024). Radiation dose optimization. https://www.medicalimaging.org/medical-imaging/principle-details/radiation-dose-optimization.

[28] Willemink MJ, de Jong PA, Leiner T, de Heer LM, Nievelstein RA, Budde RP, Schilham AM (2013). Iterative reconstruction techniques for computed tomography part 1: technical principles. Eur Radiol.

[29] Namasivayam S, Kalra MK, Pottala KM, Waldrop SM, Hudgins PA (2006). Optimization of Z-axis automatic exposure control for multidetector row CT evaluation of neck and comparison with fixed tube current technique for image quality and radiation dose. AJNR Am J Neuroradiol.

[30] Sun Z (2012). Coronary CT angiography with prospective ECG-triggering: an effective alternative to invasive coronary angiography. Cardiovasc Diagn Ther.

[31] Prabsattroo T, Wachirasirikul K, Tansangworn P, Punikhom P, Sudchai W (2023). The dose optimization and evaluation of image quality in the adult brain protocols of multi-slice computed tomography: a phantom study. J Imaging.

[32] Alqahtani MM, Willowson KP, Constable C, Fulton R, Kench PL (2022). Optimization of (99m) Tc whole-body SPECT/CT image quality: a phantom study. J Appl Clin Med Phys.

[33] (2024). Justification and optimization. https://www.iaea.org/resources/rpop/resources/international-safety-standards/justification-and-optimization.

[34] Vom J, Williams I (2017). Justification of radiographic examinations: what are the key issues?. J Med Radiat Sci.

[35] Badawy MK, Anderson A (2023). Radiation protection for comforters and carers in radiology and nuclear medicine. J Med Radiat Sci.

[36] Surić Mihić M, Mestrović T, Prlić I, Surić D (2008). Importance of quality assurance program implementation in conventional diagnostic radiology. Coll Antropol.

[37] Siedband M, Balter S, Morgan T (1984). Aapm report no. 4 basic quality control in diagnostic radiology. Nippon Hoshasen Gijutsu Gakkai Zasshi.

[38] topfishdigital topfishdigital (2024). The science behind accuracy: how medical equipment calibration keeps diagnostic devices reliable. https://www.sealcalibration.co.uk/the-science-behind-accuracy-how-medical-equipment-calibration-keeps-diagnostic-devices-reliable/.

[39] Frolovs G (2024). What is medical device test equipment calibration?. SimplerQMS.

[40] (2024). Why annual equipment calibration is so important. https://www.performancehealthacademy.com/why-annual-equipment-calibration-is-so-important.html.

[41] Harmonay V (2024). Maintaining your CT scanner calibration. https://info.atlantisworldwide.com/blog/maintaining-your-ct-scanner-calibration.

[42] European Commission (2014). Radiation protection and safety of radiation sources: international basic safety standards. AGENCY.

[43] (2024). Radiation protection and safety of radiation sources: international basic safety standards. https://www.who.int/publications/m/item/radiation-protection-and-safety-of-radiation-sources-international-basic-safety-standards.

[44] (2024). International safety standards. https://www.iaea.org/resources/rpop/resources/international-safety-standards.

[45] (2024). What are the current guidelines for radiation protection?. https://ec.europa.eu/health/scientific_committees/opinions_layman/security-scanners/en/l-3/2-radiation-protection.htm.

[46] Sonawane AU, Singh M, Sunil Kumar JV, Kulkarni A, Shirva VK, Pradhan AS (2010). Radiological safety status and quality assurance audit of medical X-ray diagnostic installations in India. J Med Phys.

[47] (2024). Radiological protection of people and the environment: generic developed principles. https://www.gov.uk/government/publications/rsr-generic-developed-principles-regulatory-assessment/radiological-protection-of-people-and-the-environment-generic-developed-principles.

[48] (2024). The ionising radiation (medical exposure) regulations. https://www.legislation.gov.uk/uksi/2017/1322/made.

[49] Yu L, Liu X, Leng S (2009). Radiation dose reduction in computed tomography: techniques and future perspective. Imaging Med.

[50] Tsapaki V (2020). Radiation dose optimization in diagnostic and interventional radiology: current issues and future perspectives. Phys Med.

[51] (2024). Optimising image quality. https://www.iaea.org/topics/optimising-image-quality.

[52] (2024). 4 future technologies that will shape Radiology. https://www.diagnosticimaging.com/view/4-future-technologies-will-shape-radiology.

[53] Najjar R (2023). Redefining radiology: a review of artificial intelligence integration in medical imaging. Diagnostics (Basel).

[54] (2024). 5 emerging radiology technologies. https://www.linkedin.com/pulse/5-emerging-radiology-technologies-advanced-health-education-center-ewpmc.

[55] (2024). Emerging trends in radiology | medical imaging solutions | AMI. https://www.astermedicalimaging.com/.

[56] Delaney FT, Doinn TÓ, Broderick JM, Stanley E (2021). Readability of patient education materials related to radiation safety: what are the implications for patient-centred radiology care?. Insights Imaging.

[57] Tohidnia MR, Cheraghi Z, Zeinodini S, Veismoradi M, Najafi M (2022). Importance of informing patients in medical imaging; radiographers’ opinion. J Clin Res Paramed Sci.

[58] Ludwig RL, Turner LW (2002). Effective patient education in medical imaging: public perceptions of radiation exposure risk. J Allied Health.

[59] Jimenez YA, Wang W, Stuart K, Cumming S, Thwaites D, Lewis S (2018). Breast cancer patients’ perceptions of a virtual learning environment for pretreatment education. J Cancer Educ.

[60] Khamtuikrua C, Suksompong S (2020). Awareness about radiation hazards and knowledge about radiation protection among healthcare personnel: a quaternary care academic center-based study. SAGE Open Med.

[61] Savage K, Arif S, Smoke M, Farrell T (2015). Patient education in radiation therapy: to teach or not to teach—that is the question?. J Med Radiat Sci.

[62] Al-Othman AY, Al-Sharydah AM, Abuelhia EI (2022). Radiation dose optimization based on saudi national diagnostic reference levels and effective dose calculation for computed tomography imaging: a unicentral cohort study. Appl Sci.

[63] Thakur Y, McLaughlin PD, Mayo JR (2013). Strategies for radiation dose optimization. Curr Radiol Rep.

